# Validation and Ecological Niche Investigation of a New Fungal Intraspecific Competitor as a Biocontrol Agent for the Sustainable Containment of Aflatoxins on Maize Fields

**DOI:** 10.3390/jof8050425

**Published:** 2022-04-21

**Authors:** Giorgio Spadola, Gianluigi Giannelli, Serena Magagnoli, Alberto Lanzoni, Marco Albertini, Riccardo Nicoli, Roberto Ferrari, Giovanni Burgio, Francesco M. Restivo, Francesca Degola

**Affiliations:** 1 Department of Chemistry, Life Sciences and Environmental Sustainability, University of Parma, 43124 Parma, Italy; giorgio.spadola1@unipr.it (G.S.); gianluigi.giannelli@unipr.it (G.G.); restivo@unipr.it (F.M.R.); 2 Department of Agricultural and Food Sciences (DISTAL), University of Bologna, 40127 Bologna, Italy; serena.magagnoli4@unibo.it (S.M.); alberto.lanzoni2@unibo.it (A.L.); giovanni.burgio@unibo.it (G.B.); 3 Agrites S.r.l., 40057 Granarolo dell’Emilia, Italy; albertini.intesia@agrites.it (M.A.); riccardo.nicoli@agrites.it (R.N.); 4 Centro Agricoltura Ambiente “Giorgio Nicoli” S.r.l., 40014 Crevalcore, Italy; rferrari@caa.it

**Keywords:** *Aspergillus flavus*, intraspecific biocompetition, niche variation, aflatoxin biocontrol, maize protection

## Abstract

Crop yield and plant products quality are directly or indirectly affected by climate alterations. Adverse climatic conditions often promote the occurrence of different abiotic stresses, which can reduce or enhance the susceptibility to pests or pathogens. Aflatoxin producing fungi, in particular, whose diffusion and deleterious consequences on cereals commodities have been demonstrated to highly depend on the temperature and humidity conditions that threaten increasingly larger areas. Biological methods using intraspecific competitors to prevent fungal development and/or toxin production at the pre-harvest level are particularly promising, even if their efficacy could be affected by the ecological interaction within the resident microbial population. A previously characterized *Aspergillus flavus* atoxigenic strain was applied in two maize fields to validate its effectiveness as a biocontrol agent against aflatoxin contamination. At one month post-application, at the harvest stage, its persistence within the *A. flavus* population colonizing the maize kernels in the treated area was assessed, and its efficacy was compared in vitro with a representation of the isolated atoxigenic population. Results proved that our fungal competitor contained the aflatoxin level on maize grains as successfully as a traditional chemical strategy, even if representing less than 30% of the atoxigenic strains re-isolated, and achieved the best performance (in terms of bio-competitive potential) concerning endogenous atoxigenic isolates.

## 1. Introduction

The last decades have witnessed the evolution of classical agriculture into a modern form, thanks to new cultivation techniques, a growing use of mechanization and agronomical inputs, and the strong diffusion of crop rotations up to monoculture. Even if responding to the global food market exigencies, it did not take long before this kind of management raised concerns regarding long-term environmental sustainability. The intensification of crop production and, in turn, the use of chemical inputs to combat phytopaties and pests have been demonstrated to exert ruinous consequences to both the environment and human health. Amongst these, the reduction and simplification of agroecosystem biodiversity, increase of toxic chemical residues in the trophic chain, and occurrences of eutrophication phenomena in both fresh and salty water have been recognized [1]. Additionally, the extensive use of synthetic pesticides has allowed the rise of organisms resistant to the major active principles, given the selective pressure they have been subjected to, periodically creating the need to find new formulations to bypass the adaptation of harmful pests [2]. In response to the need for reviewing the environmental impact of agricultural production systems, in 2007, the European Network for the Durable Exploitation of Crop Protection Strategies (ENDURE) set the goal of identifying and defining crop protection strategies reliant as little as possible on the use of chemical pesticide (http://www.endure-network.eu Accessed on 10 August 2021). A few years later, in 2009, the Directive 2009/128/EC on the sustainable use of pesticides was adopted by the European Commission as an implementation of the Integrated Pest Management (IPM) that aims to grow healthy crops by minimizing the alteration of the agroecosystem and stimulating biological control mechanisms, and that became mandatory within the European Union in 2014.

The biological control of pests, and the application of biocontrol agents (BCAs) in particular, is regarded as the most efficient approach for the containment of phytopathogens with the lowest chemical input to the agro-system. With a specific mention of pathogenic fungal species, the development of BCAs against phytophagous insects, whose feeding activity can damage the plant integrity facilitating, in turn, the infection by the fungus, is widely described [3]. The presence of a variety of Lepidoptera in maize fields, such as *Sesamia cretica* (Lederer) and *Ostrinia nubilalis* (Hübner), negatively affects the quality of the kernels, that, once damaged by their larvae, are more susceptible to the attack of phytopathogenic filamentous fungi [4]. Among them, *Aspergillus flavus* is the most hazardous due to its ability to produce aflatoxins (AFs), products of the fungal secondary metabolism that have been classified by the International Agency for Research on Cancer as the most carcinogenic natural compound ever found [5]. Because of AFs’ detrimental effects on human and animal health (which include hepatocellular carcinoma events, immunosuppression, nephropathies, and acute and sub-acute toxicoses [6,7], the Commission Regulation (EU) 2019/1869 fixed to 20 ppb the limit of AFs allowed in raw feed commodities, to 2 ppb the limit for cereals and to 0.05 ppb the limit in dairy products [8]. Strategies aimed at preventing the fungus spread on the plant material both in the field and during storage can effectively reduce AFs contamination of products [9]. However, the large diffusion of aflatoxigenic species on crops, in combination with the complexity of the factors that regulate their secondary metabolism–and thus AFs biosynthesis–and the management costs, make the obtaining of food and feed commodities completely free from AFs challenging.

The use of fungicides, which could greatly limit infection events, also contributes to the increase of undesirable toxicant residues that impact product quality and harms the environment. An indirect method for preventing AFs contamination in maize crops is reducing *O. nubilalis* presence in the field. In countries where the use of genetically modified crops is forbidden, this is typically achieved by the application of common synthetic insecticides, the use of products containing the microbial agent *Bacillus thuringiensis* var. *kurstaki* Berlinier, or the exploitation of egg parasitoids belonging to the genus *Trichogramma* Westwood (Hymenoptera: Trichogrammatidae).

Nonetheless, an increase in their application would hardly be economically sustainable with unknown future consequences [10], as observed in the case of the use of pyrethroids that caused mite-onset phenomena in *Tetranychus urticae* Koch [11]. The choice of selective insecticides or biological control strategies with low environmental impact and determining the slightest disturbance of the agroecosystem trophic equilibrium is to be pursued.

Several promising and environmentally friendly approaches for limiting *A. flavus* spread and AFs contamination of crops are based on the use of different microbial organisms such as BCAs. In particular, various species of yeasts, bacteria, and fungi have been evaluated for their biocontrol potential against *A. flavus* [12,13,14]. Contrary to the exertion of the inter-specific effects of these microorganisms on the aflatoxigenic fungus, the use of atoxigenic strains of *A. flavus* for the exploitation of intra-specific biocompetition was revealed to be an effective method to control the production of aflatoxin in various crops: successful examples have been reported in spices, cotton seeds, and maize [12,15,16,17]. Due to the different national regulations about the release of genetically modified microorganisms, to date, *A. flavus* populations colonizing the natural environment are regarded as the most profitable reservoirs for selecting the strongest competitive atoxigenic strains. Additionally, considering the ecological features on which the intra-specific biocompetition could rely (for example, the adaptation to a particular niche/crop or a well-defined microbiota asset) are pointing to the use of competitive wild strains preferably isolated from the same geographical area to be treated.

The interference mechanisms of atoxigenic strains on the accumulation of AFs have not been completely clarified yet. The prevailing opinion is that they could depend on the competitive exclusion of the aflatoxin producer strains from the substrate as a result of a successful physical shift, but the competition for nutrients has also been suggested [18]; on the other hand, controversial evidence has been reported concerning the existence of “diffusible factors” released by the biocompetitor [15,19]. Nonetheless, selecting atoxigenic biocontrol strains is quite far from being easy. In fact, while many atoxigenic strains could effectively reduce AFs contamination in vitro, their effectiveness can be highly affected by various environmental factors when applied in the field [20]. Hence, even if reconstruction experiments have generally been conducted under laboratory conditions to assess the efficacy of atoxigenic strains in preventing AFs production and/or to provide preliminary indications about their performance when released on crops, *in field* experimentations must be performed to validate their suitability as bio-competitors.

In 2009, we isolated from the maize fields of the Piedmont region an *A. flavus* atoxigenic strain that was validated as an excellent biocompetitor in in vitro intraspecific competition assays and helped to clarify some key points about intraspecific competition such as (i) the importance of the inoculum concentration and timing on the competitive advantage of both strains (toxigenic and atoxigenic), (ii) the minimum effective inoculation ratio between the two strains for AF inhibition, and (iii) how the above-mentioned factors synergistically interact in the mycotoxin accumulation process [21]. A validation of this *A. flavus* wild strain as a new intraspecific fungal biocompetitor is here described, which includes the evaluation of two commonly applied insecticides (Coragen^®^ and Turex^®^) on the biocompetitor performance in vitro, the monitoring of its persistence in the resident population colonizing the treated maize fields, and the comparison of its competitive potential with the atoxigenic strains populating the relevant niche.

## 2. Materials and Methods

### 2.1. Aspergillus flavus Strains, Media, and Culture Conditions

The atoxigenic natural strain of *Aspergillus flavus* TOϕ (fungal bio-competitor, FB hereafter) used as an intraspecific biocontrol agent in the present study was isolated from kernels belonging to the maize fields of the Po Valley, Northern Italy, in 2011 [21]. The atoxigenic strain BS07, kindly provided by Prof. K. Ehrlich (Food and Feed Safety Research, USDA-ARS, Southern Regional Research Center, New Orleans, LA, USA), was used as a reference strain (negative control) in the aflatoxin accumulation assay for the chemotypization of isolates. All the *A. flavus* isolates were maintained on YES-agar medium (2% (*w/v*) yeast extract (Difco), 5% (*w/v*) sucrose (Sigma), and 2% (*w/v*) agar (Difco)); conidia suspensions were obtained from YES cultures incubated at 28 °C in the dark up to 14 days, and conidia concentration and viability were determined according to Degola et al. [21]. *A. flavus* population from maize kernels was isolated on Dichloran Rose-Bengal Chloramphenicol Agar Base (DRBC; Oxoid Ltd., Basingstoke, UK) following manufacturer instructions. Coconut milk-derived medium (CCM) used for aflatoxin accumulation assay was obtained as described in Degola et al. [22]: briefly, 400 mL of commercial coconut cream was diluted with bidistilled water to the final volume of 1.2 L, sterilized by autoclaving, cooled at 4 °C overnight, and clarified by centrifugation. The residual floating material and the pellet were discarded, while the intermediate phase was recovered and used as a culture medium.

### 2.2. In Vitro Effect of Chemical (Coragen^®^) and Organic (Turex^®^) Formulations on A. flavus

#### 2.2.1. Effect on Germination and Early Mycelium Development

Microplates cultures in 96-well plates were set up as follows: 5 × 10^2^ conidia were inoculated in a final 200 μL YES 5% liquid medium, amended with 50 and 100 μg/mL of Coragen^®^ (chlorantraniliprole 200 g/L; Cheminova Agro Italia S.r.l., Bergamo, Italy) or Turex^®^ (*B. thuringiensis* kurstaki-HD1 and *B. thuringiensis* Aizawa-H7 25.000 U.I./mg; SCAM Spa, Modena, Italy). Optical density was recorded at 620 nm for each well with a microplate reader (TECAN SpectraFluor Plus, Männedorf, Switzerland) for 48 h of growth without shaking, in the dark, at 28 °C. Density values at 48 h were normalized by subtracting values as measured immediately after the inoculum. Samples were inoculated in quadruplicate, and experiments were conducted in triplicate.

#### 2.2.2. Effect on FB Radial Growth

A volume of 10 μL of a FB conidial suspension (10^4^ spores/mL) was point-inoculated, in triplicate, in Petri dishes containing 5% YES medium; suspensions were amended with 100 μg/mL Coragen^®^ (Cheminova Agro Italia S.r.l., Bergamo, Italy) or Turex^®^ (SCAM Spa, Modena, Italy) alternatively. Volumes of equally concentrated insecticide formulations were spotted as a negative control, and inocula on FB conidial suspension alone represented the positive control. Plates, inoculated in triplicate, were incubated at 25 °C for up to four days in the dark, then the radial growth of colonies was visually evaluated.

#### 2.2.3. Effect on Kernels Infection In Vitro

Samples of maize kernels (30 g, corresponding to about 90 seeds), previously sterilized by autoclaving, were soaked in 18 mL of a sterile, bidistilled H_2_O solution amended with 100 and 1000 μg /mL Coragen^®^ (Cheminova Agro Italia S.r.l., Bergamo, Italy) or Turex^®^ (SCAM Spa, Modena, Italy) and 10^6^ spores/mL of FB. After 5 min, kernels were set to dry and incubated in Petri dishes. The rate of seeds infection was evaluated after six days at 28 °C. Samples were prepared in triplicate, and experiments were conducted in triplicate.

### 2.3. Study Sites, Experimental Design, and Field Management

Two different experiments were conducted in two maize fields located in the province of Bologna (Italy). Seeds were sown in April. Gallup biograde^®^ 360 (glyphosate 360 g/L, at field dose of 4 L/ha; Barclay Chemicals Manufacturing Ltd., Dublin, Ireland) and Adengo^®^ (thiencarbazone-metyl 20 g/L, isoxaflutole 50 g/L, cyprosulfamide 33 g/L, at field dose of 2 L/ha; Bayer CropScience Italia, Milano, Italy) were spread on the soil as pre-emergence herbicide treatment. Pre-sowing fertilization was conducted with Nutrifos Hp (300 kg/ha; SCAM Spa, Modena, Italy); coverage nitrogen fertilization was performed with urea (46% N; 500 kg/ha).

#### 2.3.1. Study Site #1: Maize Variety and Aflatoxin Control Strategies Application

Maize hybrid DKC60-40 (Decal, Monsanto) was used. Three plots (200 m × 20 m) were divided into 4 randomized sub-plots (1000 m^2^) each; a buffer zone of about 10 m was set up between the plots, and two treatments were performed as follows: (1) conventional chemical strategy, based on a single application of Coragen^®^ (Cheminova Agro Italia S.r.l., Bergamo, Italy) at the field dose of 125 mL/ha in 2 hL/ha of water (control); (2) intraspecific biocompetition and chemical strategy combined: based on the application of a FB conidial suspension (at the field dose of 2.5 × 10^11^ conidia in 250 mL, in 2 hL/ha of water) mixed with Coragen^®^ (Cheminova Agro Italia S.r.l., Bergamo, Italy) at a field dose of 125 mL/ha in 2 hL/ha of water. Treatments were sprayed on plants by an air-assisted sprayer (Model Gaspardo Uragano 3000) in the first week of July, as the second flight of *O. nubilalis* has recorded. Control plots were not subjected to any AFs control strategy.

#### 2.3.2. Study Site #2: Maize Variety and Aflatoxin Control Strategies Application

Maize hybrid Pico AMERICAN^®^ genetics was used. Four plots (200 m × 20 m) were divided into 4 randomized sub-plots (1000 m^2^) each; a buffer zone of about 10 m was set up between the plots, and three treatments were performed as follows: (1) conventional chemical strategy, based on a single application of Coragen^®^ (Cheminova Agro Italia S.r.l., Bergamo, Italy) at the field dose of 125 mL/ha in 2 hL/ha of water; (2) intraspecific biocompetition strategy: based on the single application of a FB conidial suspension at a field dose of 2.5 × 10^11^ conidia in 250 mL in 2 hL/ha of water; (3) biological conventional strategy, based on a single application of Turex^®^ (SCAM Spa, Modena, Italy) at a field dose of 1 kg/ha in 2 hL/ha of water. Treatments were applied in the first week of July, as the second flight of *O. nubilalis* was recorded. Control plots were not subjected to any AFs control strategy.

#### 2.3.3. Maize Harvesting and Samples Collection

Maize was mechanically harvested in August. Incremental amounts of maize grains (100 g each) were randomly and continuously collected during harvest, up to approximately 5 Kg for each sample (four per strategy). A sub-sample of 1 Kg from the 5 was addressed to aflatoxin B_1_ (AFB_1_) determination by high-pressure liquid chromatography (HPLC). Another sub-sample of 500 g from each sub-plot addressed *A. flavus* strains population isolation and characterization.

### 2.4. Aflatoxin B_1_ Dosage in Maize Kernels

The AFB_1_ determination was carried out by high-pressure liquid chromatography (HPLC), as reported by Magagnoli et al. [23] following the Kobra^®^ Cell method (R-Biopharm Rhone, Ltd., Glasgow, UK). Briefly: 20 g of maize kernels were grounded in a blender with 100 mL 70% MetOH, filtered through a paper filter, diluted 1:5 in Milli-Q water, and filtered with a microfiber filter (1.5 μm, VICAM, Watertown, MA, USA). Ten milliliters of filtrate were passed through the immunoaffinity clean-up column (Afla B&G, ORSELL, Modena, Italy). AFs were recovered by washing the column with 1.5 mL CH_3_OH; finally, 0.5 mL of Milli-Q water was added to the flow-through. The chromatographic analyses were performed using a Jasco Model PU-1580 pump, equipped with a Hypersil™ ODS C18 column (250 mm × 10 mm, Thermo-Fisher Scientific, Waltham, MA, USA), a Jasco Model AS-1555 autosampler (loop = 0.1 mL), and a Jasco Model FP-1520 fluorescence detector (λ_ex_ = 365 nm and λ_em_ = 440 nm). Run conditions were as follows: mobile phase Water:Acetonitrile: Methanol (72:14:14 *v/v/v*) with nitric acid and KBr for KOBRA cell (injection volume 400 μL; flow rate 1.2 mL/min).

### 2.5. Isolation and Characterization of the Resident A. flavus Population

#### 2.5.1. Strains Isolation and Chemotypization

The resident population of *A. flavus* was isolated from sampled maize kernels. Seeds from each sub-plot were washed with a sterile washing solution (0.1% Tween20 in double-distilled water) and plated on a DRBC selective medium (100 μL of a 1:10 dilution). Plates were incubated at 31 °C in the dark for three days. Plates with heavy yeast contamination were discarded. For each parcel, 30 valid plates were obtained for 120 plates/treatment. Colonies classified as *A. flavus* according to Pitt and Hocking [24] and Samson et al. [25] were recovered, up to three from each plate. Strains were then single-spore re-isolated and assayed for AF accumulation capacity, according to Degola et al. 2012 [22], to be divided into aflatoxigenic (afla+) and atoxigenic (afla-) strains.

Suspensions of spores from YES cultures were diluted in CCM and brought to the final concentration of 5 × 10^2^ conidia/well in a final volume of 200 µL/well, in standard flat-bottom 96-well microplates (Sarstedt, Newton, NC, USA). Plates were incubated in a static condition, in the dark at 25 °C, for six days. Readings were performed directly from the bottom of the culture plate with a fluorescence microplate reader (TECAN SpectraFluor Plus, Mannedorf, Switzerland; λ_ex_ = 360 nm; λ_em_ = 465 nm; manual gain = 83; lag time = 0 µs; number of flashes = 3; integration time = 200 µs). Fluorescence values were normalized by subtracting the values of a control culture of the atoxigenic strains BS07. Each strain was inoculated in triplicate.

#### 2.5.2. Molecular Characterization of *A. flavus* Strains

A rapid method for gDNA extraction was used for all the strains isolated: mycelia from multi-well microplates of aflatoxin production assay were recovered, frozen in liquid nitrogen and, using a pestle, ground to a powder. Then, 400 μL of lysis buffer (EDTA 50 mM, SDS 0.2%, pH 8.5) were added to the samples and gently mixed. After 10 min at room temperature, samples were centrifuged at 15,000 rpm at 4 °C for 15 min; then 62.5 μL of 3M CH_3_COONa were added to the recovered supernatant, mixed by inversion, maintained at 4 °C for 60 min, and centrifuged at 15,000 rpm at 4 °C for 15 min. The pellet was discarded while the supernatant, containing the gDNA, was then diluted 1:50 in ultrapure water, quantified with a NanoDrop ND-1000 (NanoDrop Technologies, Wilmington, DE, USA) and stored at −20 °C or immediately used as a template in a Random Amplification of Polymorphic DNA (RAPD-PCR) analysis.

RAPD-PCR was performed in 20 μL of PCR Flexi Buffer 5× (Promega Corp., Madison, CA, USA), containing 1 μg of gDNA, 25 mM MgCl_2_, dNTPs 25 mM, 10 μM of the degenerated oligonucleotide 5′-GAGAGAGAGAGAGAGAYG-3′ and 0.5 U of GoTaq^®^ DNA-polymerase (Promega Corp., Madison, CA, USA). The cycling parameters were: 4 min at 94 °C; for 35 cycles: 1′ at 94 °C, 20 sec at 44 °C, and 2 min at 72 °C; final extension for 6 min at 72 °C. Amplification patterns were visualized on 2% agarose gel, while the amplification bands number and distribution were analyzed using Quantity One^®^ software version 4.6.6 (Bio-Rad Laboratories, Inc., Hercules, CA, USA).

#### 2.5.3. In Vitro Bio-Competition Assay

The bio-competition assay was carried out using the multi-well microplate fluorescence-based procedure, according to Degola et al., 2011 [21]. Suspensions of spores from both afla+ and afla- strains were co-inoculated in a 200 μL final volume of CCM (5 × 10^2^ conidia/well for each strain) to obtain a final concentration of 10^3^ conidia/well. Wells inoculated with 5 × 10^2^ conidia/well of single strains served as control. Plates were incubated at 25 °C for six days. Aflatoxin accumulation in culture wells was measured with a fluorescence microplate reader (TECAN SpectraFluor Plus, Mannedorf, Switzerland; λ_ex_ = 360 nm; λ_em_ = 465 nm; manual gain = 83; lag time = 0 µs; number of flashes = 3; integration time = 200 µs). Fluorescence values were normalized by subtracting the values of the single strain atoxigenic cultures. The competitive activity of afla- strains was expressed as percentage inhibition of AF production concerning the relevant afla+ cultures. Cultures were inoculated in quadruplicates, and experiments were performed in triplicate.

### 2.6. Statistical Analyses

Statistical analyses were performed with the Past 3.x software [26]. For AFB_1_ quantitation and hyphae early growth data, one-way analysis of variance (ANOVA) was used; for relative abundance and aflatoxin inhibition data, analysis of variance was performed by the Levene test and then the Kruskal–Wallis test was performed. Differences were considered statistically significant at a *p* < 0.05.

## 3. Results

### 3.1. Coragen^®^ and Turex^®^ In Vitro Effect on the Fungal Bio-Competitor (FB) Fitness

**Figure 1:**
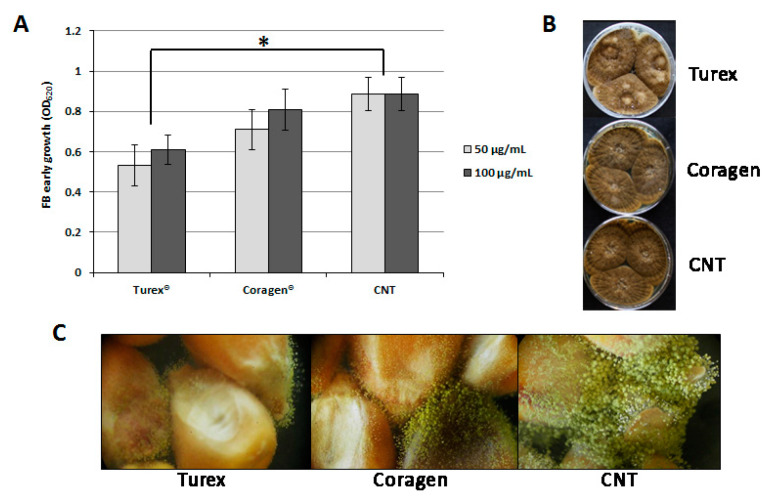
Assessment of insecticide formulations’ effect on FB growth, development, and colonization ability. (**A**): Conidia germination and hyphae early growth evaluation in a multi-well microculture system, in YES liquid medium, with 5 × 10^2^ FB conidia in the presence of 50 and 100 μg/mL Coragen^®^ or Turex^®^ alternatively. Data reported as mean ± S.D. Asterisk indicates significant differences at *p*-value < 0.01. (**B**): FB radial growth visual evaluation in YES solid medium; 10 µL of a mixture of FB conidial suspensions (5 × 10^2^ conidia) and 100 μg/mL of Coragen^®^ or Turex^®^ alternatively were point inoculated, in triplicate, in YES 5% agar plates; control (CNT) was represented by 10 µL of FB conidial suspensions (5 × 10^2^ conidia) without insecticide amendment. (**C**): FB colonization of maize kernels surface-treated with FB conidial suspensions (5 × 10^2^ conidia) amended with 100 μg/mL Coragen^®^ or Turex^®^ alternatively; control (CNT) was represented by maize kernels treated with FB conidial suspensions (5 × 10^2^ conidia) without insecticide amendment.

With a perspective of desirable exploitation of the synergism between commonly applied insecticides and the FB in reducing AF grains contamination, the possible effect of Coragen^®^ and Turex^®^ products on the growth of the FB and its ability to colonize corn kernels was evaluated (Figure 1). *A. flavus* micro-cultures were set up in 96 multi-well plates with YES liquid medium amended with two concentrations of Coragen^®^ and Turex^®^. Each well was inoculated with fungal conidia to measure the effect of insecticides on the conidia germination and mycelium early development. The effect of Coragen^®^ and Turex^®^ on the radial growth of FB colonies was also investigated in Petri dishes of YES solid medium added with the same concentrations of insecticide, and the ability to colonize maize kernels surface-treated with Coragen^®^ or Turex^®^ was assessed.

The Turex^®^ formulation was found to affect the early growth of FB mycelium slightly, which was probably delayed by the presence of *B. thuringiensis* and its Δ-endotoxin; on the contrary, Coragen^®^ did not exert any inhibitory activity at this developmental phase (Figure 1A). When mixed to the conidial suspension and spotted on a solid medium, both products were ineffective in limiting the radial growth of FB colonies, which didn’t show visible differences concerning the control in terms of either colony diameter or conidiation rate (Figure 1B). Different results were found, instead, by observing how the FB reached to colonize the surface of kernels treated with the two insecticides: both treatments finally resulted in successful fungal colonization, but to a different extent, as Turex^®^-treated seeds showed a less consistent and developed mycelium (Figure 1C).

### 3.2. In Field Validation of the FB Effectiveness against AF Contamination

The efficacy of our *A. flavus* wild strain as a biocontrol agent for the containment of AF contamination on maize kernels was assessed in two different study sites (Table 1); agronomical practices applied in the management of the site were to maximize plant health. In study site #1, where the FB treatment was compared in terms of AFB_1_ reduction with a Coragen^®^ application, the biocompetition strategy resulted in the complete avoidance of toxin contamination of maize kernels. At the same time, the conventional chemical method was found to lower the AF concentration from 1.20 ppb, as measured in the control samples, to 0.24 ppb. Study site #2 compared the intraspecific biocompetition strategy with both the conventional chemical method and the conventional biological approach exerting the direct antagonist effect of *B. thuringiensis* on corn borers (Turex^®^). As reported in Table 1, the Turex^®^ application did not effectively reduce AF contamination; on the contrary, Coragen^®^ and FB applications significantly affected the toxin level on treated plots, even if to different extents.

### 3.3. Characterization of the Resident A. flavus Population and Persistence of the FB after the Application

**Figure 2:**
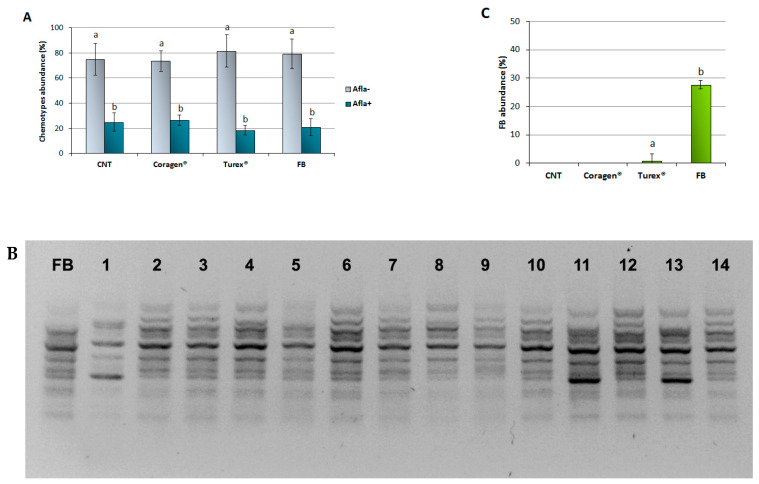
Characterization of the *A. flavus* population at the moment of the harvest. (**A**): Relative abundance of the two chemotypes (afla+ and afla-) colonizing the maize kernels from the different treatments. Data, expressed as the percentage of *A. flavus* strains total number isolated in each treatment, are reported as mean ± S.D. Different letters indicate significant differences at *p*-value < 0.01. (**B**): Example of the molecular profiling of afla- isolates used to track the FB persistence on the crop. (**C**): FB persistence within the afla- *A. flavus* population. Data, expressed as the percentage of the afla- *A. flavus* strains total number isolated in each treatment, are reported as mean ± S.D. Different letters indicate significant differences at *p*-value < 0.05.

Post-harvest analyses were conducted on maize kernel samples from study site #2 to isolate the resident population of *A. flavus*. Strains were isolated from harvested grains and characterized at the molecular level to both evaluate the persistence of the bio-competitor and to compare the aflatoxigenic/atoxigenic strains ratio in the different treatments. A total of 1500 isolates were recovered and classified for AFs production. We found that afla- strains prevailed, in terms of abundance, over the afla+ population (73–81% versus 18–26%, respectively); interestingly, this prevalence was consistently observed in all the treatments, without exceptions (Figure 2A).

All the atoxigenic strains were then genotyped to track and quantify the FB presence in the afla- population. A RAPD-PCR was used that was particularly effective in discriminating *A. flavus*, strains able to produce a unique amplification pattern for the FB concerning all afla- isolates. An example is shown in Figure 2B, where the FB electrophoretic profile is compared with the profile of 14 isolates; in this case, none of the samples were classified as a re-isolation of FB from treated maize. The molecular characterization of isolates was prodromal for the subsequent evaluation of FB abundance within the atoxigenic population colonizing the experimental field: as expected, the biocompetitor was found in the relevant treated parcel, representing about the 27% of the afla- *A. flavus* strains (Figure 2C); however, an occasional presence was disclosed in the Turex^®^ treated parcel also, that was probably due to sporadic contamination occurred during the FB administration in the adjacent parcel. On the contrary, neither in the control nor in the chemical strategy has the re-isolation of the bio-competitor been encountered.

### 3.4. Comparative Evaluation of the FB Competitive Ability against the Resident A. flavus Population

**Figure 3:**
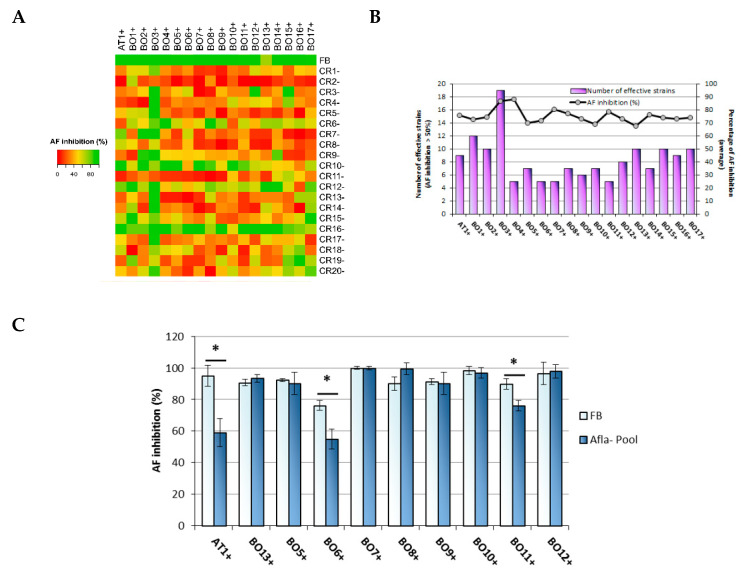
In vitro bio-competition challenge tests of the FB against the toxigenic *A. flavus* population colonizing the study site #2. (**A**): Heat map built with bio-competition efficacy of 20 afla- against 17 afla+ isolates. Conidia were co-inoculated in a 1:1 ratio. AT1+ strain was used as reference strain; data are expressed as a percentage of AF inhibition. Map was built with the Heatmapper online tool [27]. (**B**): Number of afla- strains effective in lowering AF production (inhibition > 50%) for each afla+ isolate plotted against the average of AF inhibition determined by effective bio-competitors. (**C**): Comparison of the bio-competitive efficacy of the FB and ten afla- isolates, pooled together, against ten afla+ strains. Data reported as mean ± S.D. Asterisk indicates significant differences at *p*-value < 0.01.

A deeper dissection of the bio-competitive behavior of the FB was performed through the evaluation of the endemic *A. flavus* population’s responsiveness to the competitor’s activity (Figure 3). Hence, a battery of in vitro co-inoculation trials was set on a representative selection of isolates; a high-throughput procedure was used to assess the effectiveness of the FB to contain AFs accumulation by toxigenic strains [22]. At first, 37 strains (17 afla+ chosen amongst high-level aflatoxin producers and 20 afla- randomly selected) were employed to perform a “global” bio-competition assay: each afla- strain was challenged with each afla+ isolate and compared with the inhibitory FB’s performance. As shown in Figure 3A, the containment effect of FB on toxin accumulation was confirmed as the best, that reached inhibition levels exceeding the 85% in almost all afla+ isolates. However, when individually evaluated, a few afla- isolates resulted in an inhibition rate against specific afla+ strains higher than FB, as in the case of CR16- that lowered AF accumulation by BO13+ more efficiently (100% vs. 70% respectively), or CR6- concerning BO8+ (99% vs. 90%). On the other hand, if we consider the threshold of 50% AF inhibition, it can be observed that some afla+ isolates were more prone to be inhibited than the others, such as BO1+ and BO3+. In fact, even if they were strong AF producers, these two strains were reduced in toxin accumulation by the highest number of afla- isolates (Figure 3B). Based on a 50% cut-off, most toxigenic strains were inhibited by less than ten atoxigenic isolates amongst the 20 selected for the challenge test. In this condition, the average toxin abatement obtained for each afla+ strain with the battery of afla- isolates that were competitively effective ranged from a minimum of 67.7 to a maximum of 88.4%.

A second challenge assay was conducted to compare the effectiveness of the FB with a conidial mixture obtained by pooling conidial suspensions from the best performing afla- resident isolates. Ten atoxigenic strains were chosen amongst those that proved to inhibit the AF production of more than 50% in the highest number of toxigenic isolates (namely, CR1-, CR4-, CR6-, CR10-, CR12-, CR15-, CR16-, CR18-, CR19-, and CR20-). The efficacy of the toxin containment of both FB and the pool was tested against ten afla+ strains, nine isolated from the experimental field, and the reference strain AT1+. Results, reported in Figure 3C, showed that the afla- pool generally reached the inhibition percentage of the FB, except for three afla+ isolates: in fact, and surprisingly, the containment activity on AF accumulation of the pool against AT1+, BO6+ and BO11+ was significantly lower.

## 4. Discussion

The geographical distribution of economically important crops potentially affected by aflatoxin contamination is rapidly evolving, mainly due to the climate change and pesticide resistance phenomena [28]; for example, the AF global occurrence in South-East Asia increased from 43% to 51% from 2016 to 2020, while in Central America occurrence rose from <1% to 14% [29]. Even if AF diffusion on susceptible crops (such as maize, groundnut, cottonseed, and others) occurred more frequently in tropical and subtropical regions [30,31], the European area and many developed countries are currently interested in the high risk of exposure, as reported several years ago by Food Security Authorities [32,33]. The application of atoxigenic strains of *A. flavus* to reduce aflatoxin contamination has been successfully exploited on different crops in the USA, Africa, and recently in Italy [34,35,36]. The efficacy of this biological control strategy, based on the use of atoxigenic *A. flavus* strains naturally occurring and thus considered well adapted to the ecological niche of application, has been attributed to the displacement of the aflatoxin-producing strains. However, other interacting factors still need to be investigated since the fitness of the fungus as an effective intraspecific bio-competitor could depend not only on several environmental features (i.e., association with the crop, intra- and interspecific competition with other microorganisms, temperature, humidity) to which it might be better adapted, but also on innate, strain-specific biological characteristics that should be assessed. Studies performed in vitro previously showed that, even if both timings of afla- inoculation and afla-/afla+ inoculum ratio are crucial parameters concerning the efficacy of AF containment during bio-competition, the relative abundance of afla+ and afla-strains in terms of conidia representation in co-inoculation experiments was proved to be equal to the initial inoculum [21]; this suggests that competition for nutrients and space might not be the sole factor involved in the AF inhibition. In addition, a correlation between the inhibition rate of toxin accumulation during bio-competition and the relative abundance of the producing mycelium has never been reported, while the AF reduction is, in most cases, higher than expected by a competitive exclusion itself.

In our study area, the application of TOϕ strain (FB) proved to be as effective as the chemical treatment against corn borer (Coragen^®^) in containing the AF contamination of maize grains, being also more efficient than an organic strategy based on *B. thuringiensis* (Turex^®^). The analysis of the *A. flavus* population at the harvest showed that the intraspecific biocontrol treatment didn’t alter the afla+/afla- ratio on grains (about 20/80%), in accordance with a recent work that reported a field displacement of aflatoxigenic strains in the soil-but not at the kernels level-in response to the application of three *A. flavus* biocontrol strains [37]. Authors found a highly variable percentage of presumptive toxigenic strains during four years of experimentation, that ranged from 50 to 10%, but did not claim any significant effect of the treatment on the AF concentration in the harvested corn because of the scarce number of contaminated samples, probably due to the unfavorable climatic conditions that naturally kept low the level of mycotoxins contamination. However, the molecular characterization of afla- isolates conducted in our study highlighted that the FB represented 27% of the atoxigenic population colonizing grains from treated plots. Thus, a displacement of resident afla- strains within their population rather than a displacement of toxigenic strains seemed to occur. Like Weaver and Abbas, other authors suggested that a modified afla+/afla- ratio is the cause of AFB reduction obtained after a successful application of atoxigenic *A. flavus* bio-competitors [36]; nonetheless, our findings are in contrast with this hypothesis, since we obtained an efficient aflatoxin containment without any alteration of chemotypes ratio of *A. flavus* population. Any significant diffusion of the FB toward the areas subjected to different treatments has been observed; therefore, the sporadic isolation of TOϕ strain in grain samples from the Turex^®^ treated plots should be attributed to occasional events of passive movement of inoculum, as was widely reported in the literature.

The influence of genetic structure shifts in *A. flavus* indigenous soil populations on intraspecific biocontrol efficacy has also been investigated concerning the application of biocontrol products Afla-Guard^®^ [38] and AF36 [39] in maize crops, as the persistence in the soil of biocontrol agents is thought to offer a better efficacy in reducing aflatoxin accumulation over several generations of *A. flavus*. Unfortunately, the low levels of AFs contamination detected did not directly correlate the shift in the genetic structure of the *A. flavus* population with the toxin amount [17]. Interestingly, while most *A. flavus* isolates recovered after applying treatments belonged to the same multilocus haplotype of Afla-Guard^®^, only very few shared the same multilocus haplotype of the AF36 strain. Once again, this observation supports the idea that rather than the persistence of the biocompetitor, its effectiveness relies more on its adaptation to the *A. flavus* agroecological niche insisting on the application area.

Regardless of the biological mechanism that governs the aflatoxin inhibition in afla+ strains, it is worth noting that the tendency in being contained by several isolates is strictly strain-dependent: for example, strain BO3+ was the toxigenic isolate that was affected more than others in the toxin biosynthesis in terms of atoxigenic effective bio-competitors; this predisposition correlates with the average of AF inhibition, that was almost the highest recorded and reached about 90%. However, the observation that BO4+ showed the same containment level but exerted by the lowest number of effective atoxigenic strains shed a different light on the nature of this behavior. Similarly, some afla- bio-competitors–among them our FB–proved effective against a huge number of afla+ isolates, while, in the same conditions, others were useless (as in the case of CR2-, CR5-, CR7-, and CR8-). These competitive differences suggest that a more complex interplay than a one-to-one interaction, based on the inoculum abundance and environmental conditions, might play a role in the bio-competition performance. Additionally, the assumption that a correlation between mycelium colonization ability and spore production by two strains, competing for the same substrate is the winning strategy exploited by atoxigenic strains to prevail over competitors in the occupation of the niche [18] has not been supported by our in vitro challenge tests, where the competitive aptitude of each strain was assessed in conditions that do not depend on the conidia production process.

Despite the evidence that only a few atoxigenic strains can exert an inhibitory effect on AF accumulation by afla+ isolates, the very fact that such particular competitors can be isolated within the *A. flavus* population colonizing maize fields should be a clue of their ability to persist together with aflatoxigenic strains in the relevant crop environment, that is considered one of the key features essential for efficient biocontrol of aflatoxin contamination. Thus it was not surprising that three afla- strains (namely CR6-, CR10-, and CR16-) were highly effective against almost all afla+ isolates from the *A. flavus* resident population to which they belong. On the other hand, the best performer in terms of AF containment level and number of contained strains remained TOϕ, which was isolated many years ago from the population colonizing a very distant agroecological area (province of Turin, Piedmont), suggesting that regarding the bio-competitive ability more than a simple “niche adaptation” mechanism. It is also to be considered that afla+ strains isolated from the same area of the best bio-competitors are probably the most adapted to share the niche with such atoxigenic strains. Hence, it should be kept in mind when using them as biocontrol agents for AF reduction in the field [40]. The approach to pool conidia from the most effective bio-competitors that intuitively might overcome this issue provided interesting findings: when challenged against single afla+ strains, afla-pool was as effective as the FB alone in most cases, with few exceptions. However, this could not be exclusively attributed to isolates belonging to the same population since a significantly lower efficacy has been recorded against both inner (BO6+ and BO11+) and outer (AT1+) afla+ strains.

Here, once again, we found the suggestion that another strain-dependent feature is involved in the intraspecific interaction governing the AF biosynthesis containment, which has still to be unraveled.

## 5. Conclusions

As a pre-harvest sustainable strategy for controlling AF contamination, the application of *A. flavus* atoxigenic strains has been extensively supported and validated in various Countries. Besides its innate competitive ability, each “good bio-competitor” should be selected to efficiently interact with the fungal population of the area of application, to which it supposedly has to establish a colonization advantage not necessarily in terms of spatial occupation. On the other hand, our results showed that an efficient biocontrol agent found outside the resident *A. flavus* population successfully contained AF on maize from the first application. Because this non-native strain didn’t have time to acquire the potential to establish itself in the new environment permanently, and it did not significantly alter the composition of the inhabitant *A. flavus* population, its effectiveness must rely on peculiar competitive interactions with resident strains, interactions worthy of further investigations to obtain the formulation of the best bio-pesticide. In addition, and as suggested in 1987 by Ehrlich [41], we proved the efficacy of a multi-competition approach, such as the use of a pool composed of the most effective afla- strains, that improved the competitive performance of the single isolates to interfere with aflatoxin production by a higher number of afla+ strains.

Even if scarcely feasible from an economic point of view, further surveys for the validation of specific bio-competitors from different areas could provide an updated map of the interacting *A. flavus* populations, upgrading the panel of the “good competitors” as they will appear due to evolving agroecosystemical conditions. In fact, besides the importance of evaluating the intraspecific biocontrol agents’ efficacy case-by-case, it is manifest that the population ecology of *A. flavus* in the application area is worthy of consideration, as it will play a critical role in the design of successful biocontrol strategies in maize crops. In this sense, the use of locally or regionally adapted atoxigenic biocompetitors is recommended, as their performance could be favored by the establishment of more functional ecological interaction with indigenous aflatoxigenic strains, which result in a more efficient and less perturbing biocontrol strategy.

## Figures and Tables

**Table 1:** Reduction of AF contamination obtained with conventional chemical method (Coragen^®^), conventional biological method (Turex^®^), and biocompetition strategy (FB). AFB_1_ was HPLC-measured in maize kernels sampled from treated parcels and non-treated parcels (CNT); values are expressed as a mean of 4 replicates per treatment ± S.D. Limit of detection (LOD) value < 0.05 ppb. Asterisk indicates significant differences regarding the control at *p*-value < 0.05.

	Treatment	AFB_1_ (ppb)
Study site #1	CNT	1.20 ± 0.09
	Coragen^®^	0.24 * ± 0.05
	Coragen^®^ + FB	<LOD *
Study site #2	CNT	66.51 ± 16.87
	Coragen^®^	29.25 * ± 15.36
	Turex^®^	45.70 ± 23.67
	FB	17.86 * ± 10.57

## Data Availability

*A. flavus* strains used in this work strains are available, under request, by the Authors.
